# Prevalence of multiple human intestinal parasites across diverse environments in Madagascar

**DOI:** 10.1371/journal.pntd.0014380

**Published:** 2026-06-04

**Authors:** Stephanie M. Wu, Sarina S. Gupta, M. Ando Ravelomanantsoa, Santino Andry, Daniel F. Viana, Jessica Zamborain-Mason, Uwajachukwumma A. Uzomah, Hervet J. Randriamady, Chase Howard, Graham Friedman, Amanda Castonguay, James Hazen, Danny A. Milner, Benjamin L. Rice, Christopher D. Golden

**Affiliations:** 1 Division of Psychiatry, University College London, London, United Kingdom; 2 Department of Psychology, Western Michigan University, Kalamazoo, Michigan, United States of America; 3 Institut Malgache des Vaccins Vétérinaires, IMVAVET, Antananarivo, Madagascar; 4 Department of Entomology, University of Antananarivo, Antananarivo, Madagascar; 5 Minderoo Foundation, Perth, Western Australia, Australia; 6 Department of Nutrition, Harvard TH Chan School of Public Health, Boston, Massachusetts, United States of America; 7 Biological and Environmental Science and Engineering Division, King Abdullah University of Science and Technology, Thuwal, Saudi Arabia; 8 Lancaster Environment Centre, Lancaster University, Lancaster, United Kingdom; 9 Department of Radiation Oncology, University of Iowa, Iowa City, United States of America; 10 Harvard College, Cambridge, Massachusetts, United States of America; 11 Department of Microbiology, Brigham and Women’s Hospital, Boston, Massachusetts, United States of America; 12 Catholic Relief Services, Baltimore, Maryland, United States of America; 13 Department of Pathology, Olive View-UCLA Medical Center, Sylmar, California, United States of America; 14 Department of Ecology and Evolutionary Biology, Princeton University, Princeton, New Jersey, United States of America; Wellcome Sanger Institute, UNITED KINGDOM OF GREAT BRITAIN AND NORTHERN IRELAND

## Abstract

**Background:**

Intestinal parasitic infections affect more than 1·5 billion people globally, leading to severe health consequences such as malnutrition, anemia, diarrhea, and impaired cognitive development.

**Methodology/Principal findings:**

Samples were collected from 3,872 individuals (all ages and both sexes) across 31 rural communities in Madagascar between 2013 and 2017, representing diverse ecological and socioeconomic regions. Intestinal parasite prevalence was assessed by fecal microscopy. Bayesian multilevel logistic regression models were used to estimate overall and regional prevalences while accounting for demographic and spatial variability.

Parasite prevalence varied widely across Madagascar, with the highest rates observed for *Ascaris lumbricoides* (22·0%) and *Trichuris trichiura* (15·3%), followed by *Hymenolepis nana* (up to 10·5%), hookworm (up to 8·1%), *Strongyloides* (0·5%), and *Schistosoma mansoni* (0·5%). Infection burden was greatest in the northeast and southeast—especially among school-aged children aged 5–19. Sex differences were minor, except for higher hookworm prevalence in males.

**Conclusions/Significance:**

This study provides the most comprehensive assessment to date of intestinal parasite prevalence across Madagascar, revealing that *A. lumbricoides* and *T. trichiura* infections were highly endemic in the humid eastern regions, while *H. nana* was most common in dry regions. The findings highlight substantial geographic heterogeneity and underscore the need for regionally targeted, multi-sectoral interventions, including improved sanitation and deworming.

## Author summary

Intestinal parasitic infections affect more than 1.5 billion people worldwide and are especially burdensome in low- and middle-income countries such as Madagascar. Prevalence across Madagascar is high but varies greatly by region. In humid eastern and southeastern rainforest areas, infection rates for soil-transmitted helminths such as Ascaris lumbricoides and Trichuris trichiura often exceed 50%, while some northern and western districts report rates below 5%. Schistosoma mansoni shows similarly sharp contrasts, reaching over 70% in parts of the east but remaining uncommon in western sites.

To address fragmented and regionally inconsistent data, this large-scale study analyzed fecal samples from 3,872 individuals across 31 communities spanning five regions and four ecological zones. Overall, A. lumbricoides (22.0%) and T. trichiura (15.3%) were most prevalent, with infections highest in northeastern and southeastern rainforest regions and lowest along the west coast where *Hymenolepis nana* was most prevalent. School-aged children carried the greatest burden of soil-transmitted helminths, while hookworm was more common in males.

Persistent high prevalence—despite deworming efforts—highlights the need for geographically targeted interventions, improved water, sanitation, and hygiene (WASH) infrastructure, better medicine access, and strategies addressing upstream forest and water management, poverty, malnutrition, and climate-related risks.

## 1. Introduction

Intestinal parasitic infections affect more than 1·5 billion people globally, with the burden of disease falling disproportionately on low- and middle-income countries in tropical and sub-tropical regions such as Madagascar [1]. Heavy infections contribute to serious health problems, with soil-transmitted helminths (STH) such as *Ascaris lumbricoides*, *Trichuris trichiura*, and hookworm (*Ancylostoma duodenale* and *Necator americanus*) resulting in abdominal pain, diarrhea, and malnutrition. For example, it is well established that hookworm infections can lead to iron-deficiency anemia [2]. In children, these intestinal parasitic and pathogenic infections are particularly alarming due to associated malnutrition, stunting, and impaired growth and development [3]. Madagascar is particularly vulnerable to intestinal parasite infections due to the high levels of poverty, malnutrition, and poor sanitation [4–9].

Previous epidemiological studies have found a high burden of intestinal parasites in Madagascar, though results exhibit substantial heterogeneity due to the island’s diverse socio-ecological conditions. Prevalence is consistently high in the eastern portions of the country. In the humid coastal Vatomandry district, Scarso et al. observed high prevalence of *A. lumbricoides* (45·9%), hookworm (44·6%), *T. trichiura* (32·1%), and *Strongyloides stercoralis* (35·2%) [10]. In remote rainforest villages in the southeast, prevalence of STH infections were 71·4% for *A. lumbricoides*, 74·7% for *T. trichiura*, 33·1% for hookworm, and 3·3% for *S. stercoralis* [11]. In the eastern Marolambo district, Spencer et al. reported that over 70% of school-aged children in some villages were infected with *Schistosoma mansoni* [12]. By contrast, Richert et al. reported much lower STH prevalence (<5%) among schoolchildren in the Mampikony district in northern Madagascar [7], and prevalence of *S. stercoralis* was 5·6% in the central plateau district of Tsiroanomandidy [10]. Other surveys similarly highlight substantial heterogeneity: Rasoamanamihaja et al. (2016) reported 5·0% *S. mansoni* infection and lower prevalences of *A. lumbricoides* (4·4%), *T. trichiura* (2·2%), and hookworm (3·2%) among children aged 7–10 years in sentinel sites in the Western region of Madagascar [13].

In Madagascar, infection has also been found to be associated with demographic factors. For example, both the prevalence and intensity of *S. mansoni* infection have been observed to increase with age [12], whereas STH infections have been reported to be most common in adolescents and young children and less common in females and those with higher education [7,11]. Scarso et al. similarly found hookworm prevalence to be higher in males and those with lower educational attainment [10]. Furthermore, Krumkamp et al. reported higher schistosome infection risk among older age groups and in farmers, suggesting a potential link to occupational hazards [14]. They also observed that reduced infection risk was associated with combined higher education and knowledge of schistosome transmission, but not with either factor independently. However, findings vary across studies; Kislaya et al. reported no clear demographic correlates of *S. mansoni* infection (including sex and maternal age) among children <2 years of age [15].

Despite the high burden of intestinal parasitic infection, epidemiological data for Madagascar remain fragmented and often restricted to local studies. The high heterogeneity of ecological landscapes in the country warrants more comparable information on the prevalence of infections across varied climates and ecological zones. Prevalence of less commonly measured parasites such as *Hymenolepis nana*, *Strongyloides*, and enteric pathogens like *Entamoeba coli* also remains poorly characterized.

To address these gaps, this study investigates the prevalence and infection intensity of eight intestinal parasites and pathogens: *A. lumbricoides*, *T. trichiura*, hookworm, *Strongyloides*, *H. nana*, *S. mansoni*, other helminths, and *E. coli*. We further examined variation by age, sex, and geographic region, with the aim of identifying vulnerable demographic and geographic subgroups. The results of this analysis will inform the prioritization of medical, socioeconomic, and infrastructural interventions to reduce the burden of intestinal parasitic infections in Madagascar.

## 2. Methods

### Ethics statement

These protocols were approved by the Office for the Protection of Human Subjects at the University of California, Berkeley, CA USA (Protocol # 2007–2–3) [16] and the Committee on the Use of Human Subjects, Office of Human Research Administration at the Harvard T.H. Chan School of Public Health (Protocol #15–2230) [17] (Protocol #16–0166) [18]. The study was also reviewed and approved by the Malagasy Ministry of Health and the ethical review board at the Institut National de Santé Publique et Communautaire (INSPC) No 03/MSANP/SG/INSPC/DG/DFR. Both the HSPH IRB and the INSPC review board waived the requirement for written informed consent for this study, and approved the study’s consent procedures described previously because requiring signatures was deemed culturally inappropriate for the targeted populations.

### 2.1 Study population

Samples were collected from subjects in three studies targeting rural communities across Madagascar (Table 1): 1) a cohort study aiming to understand the importance of wild meats in human nutrition in the northeastern rainforest region near the Makira Protected Area in the Maroantsetra district with data collection occurring in 2013–2014 [16]; 2) a cohort study aiming to understand the importance of seafood in human nutrition in the northeastern littoral rainforest coastal region along the Bay of Antongil in the Maroantsetra district with data collection occurring in 2016 [17]; and 3) a cross-sectional study aiming to understand the ecological drivers of nutrition and disease spanning four regions (southeastern rainforest in the Mananjary district, southwestern spiny desert in the Toliara district, western scrubland in the Morombe district, and central plateau in the districts of Ambatofinandrahana, Fandriana, and Ambositra) with data collection occurring in 2017 [18]. All studies included both sexes and people of all ages, with households being the unit of randomization. The difference between the cohorts was centered on the geographical locations in Madagascar and the focus on different dietary archetypes. Combined, the studies included 31 communities spanning diverse social, economic, and ecological zones. All three studies used a cluster random sampling design, with households sampled from a census of all households within the 31 communities. All individuals within each selected household were enrolled and asked to provide survey data on sociodemographic variables as well as biological samples (fecal, fingernail, whole blood spots, and venous blood plasma). Complete protocol details for all three studies have been published elsewhere [16–18]. The only exclusion criteria were being too physically ill to participate. Out of a total of 3,902 participants from 1,035 households who had data available for at least one intestinal parasite, we excluded 30 (0·8%) individuals with missing data for age or sex, resulting in a total of 3,872 participants included for analysis (S1 Fig).

**Table 1:** Summary of locations, populations, and intestinal parasites assessed in Madagascar.

Region, Administrative Division, District	Number of Communities	Number of Individuals	Study Time Period	Parasites Assessed	Reference
Northeast, Analanjirofo, Maroantsetra	2	597	2013	*A. lumbricoides*, *T. trichiura*, hookworm, *Strongyloides*, other helminths, *E. coli*	16
Northeast, Analanjirofo, Maroantsetra	5	760	2016	*A. lumbricoides*, *T. trichiura*, hookworm, *Strongyloides*, *H. nana*, *S. mansoni*	17
Southeast, Vatovavy Fitovinany, Mananjary	6	730	2017	*A. lumbricoides*, *T. trichiura*, hookworm, *Strongyloides*, *H. nana*, *S. mansoni*	18
Southwest, Atsimo Andrefana, Toliara	6	662	2017	*A. lumbricoides*, *T. trichiura*, hookworm, *Strongyloides*, *H. nana*, *S. mansoni*	18
Western Coast, Atsimo Andrefana, Morombe	6	528	2017	*A. lumbricoides*, *T. trichiura*, hookworm, *Strongyloides*, *H. nana*, *S. mansoni*	18
Central Plateau, Amoron’i Mania, Fandriana	6	595	2017	*A. lumbricoides*, *T. trichiura*, hookworm, *Strongyloides*, *H. nana*, *S. mansoni*	18

**Fig 1:**
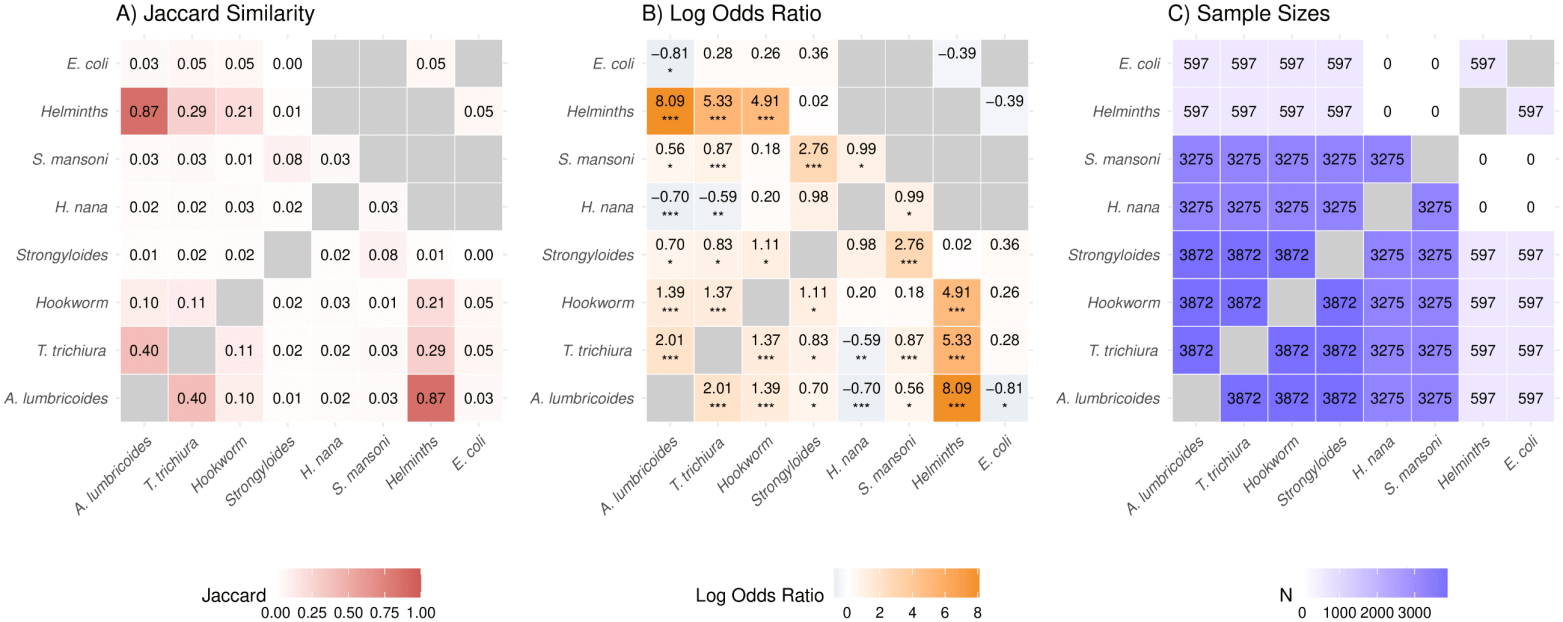
Co-occurrence of intestinal parasite prevalence. Heatmaps display measurements among pairwise complete cases: **A)** Jaccard similarity ranging from 0 to 1, quantifying overlap among positive detections; B) log odds ratio ranging from -∞ to ∞, with Fisher’s exact test significance indicated by asterisks (*: p < 0·05, **: p < 0·01, ***: p < 0·001); and C) pairwise sample size contributing to each estimate. Diagonal cells and missing pairwise estimates are shaded in gray.

### 2.2 Fecal sample collection

Each individual enrolled in the study was provided with a sterile polypropylene screw cap feces collection tube (Sarstedt, Sparks, NV; ref. 80·623). Individuals were instructed to defecate onto a clean surface (e.g., onto a waxy leaf) and then drop three small spoonfuls of feces into the tube. Informed consent was obtained from adults, verbal assent was obtained from children over 12 years of age, and permission was obtained from parents or guardians of younger children. for this study, and approved the study’s consent procedures described previously because requiring signatures was deemed culturally inappropriate for the targeted populations. Although other biological samples were collected in these studies (e.g., blood), we have only analyzed intestinal parasites from the collected fecal samples.

Once the samples were returned to the local research team (typically 10 min to 10 h after collection), 90–97% ethanol was added to the three spoonfuls of feces in a 10mL conical tube. The vast majority of samples were submitted within one hour of collection, but some were collected at night and then submitted in the morning. Samples were stored in a −23°C freezer within 14 days of collection and then shipped on dry ice and stored at −80°C at the Harvard T.H. Chan School of Public Health, where microscopic analysis was performed. In studies where stool samples were collected at multiple timepoints, the first available timepoint with parasitological results was used, and parasite values were averaged across all repeated smears for that timepoint for each individual.

### 2.3 Intestinal parasite evaluation

The presence of levels of intestinal parasites was determined from microscopic analysis of fecal samples using the formalin–ethyl acetate sedimentation concentration technique described by the Centers for Disease Control and Prevention [19].

#### 2.3.1 Sample preparation.

A predetermined subset of samples was thawed for 30 minutes under a biosafety hood. Corresponding 15 mL conical tubes were labeled with participant identifiers and recorded on standardized data sheets containing columns for sample ID, fecal weight, and 2–3 slide readings. Excess liquid was removed from each thawed specimen, and fecal weight was measured using a tared plastic cup. The amount of stool used was a mean of 0.68g (SE: 0.006g). Ten percent formalin was then added to homogenize the specimen, and additional 10% formalin was added during filtration through a gauze-lined paper cone to bring the suspension in the 15 mL conical tube to a final volume of 9 mL before centrifugation. The suspension was then centrifuged at 500 × g for 10 minutes. The supernatant was decanted, and the sediment was washed with 9 mL of 10% formalin, followed by the addition of 3 mL of ethyl acetate. After a second centrifugation under the same conditions, debris was loosened and the upper layers discarded. When debris persisted, a third centrifugation cycle was performed. The final sediment was resuspended in 1–2 drops of 10% formalin and stored with a tight seal.

#### 2.3.2. Microscopic examination.

Prior to microscopic examination, the sediment was mixed thoroughly using a disposable plastic pipette. One to three drops of the specimen were placed on a glass slide, stained with 1–2 drops of iodine, and covered with a coverslip. All researchers who read these slides were either experts (JM, DAM, AC) or trained by experts (SG, MAR, SA, UAU, CH, GF). Slide interpretation followed a staged training and quality assurance process rather than a fixed system in which every slide read by a trained reader was subsequently confirmed by an expert reader. Initially, expert readers performed slide analyses while training additional readers through direct supervision and side by side review. During the training phase, trainees and expert readers independently reviewed the same samples and compared findings. When discrepancies arose in parasite identification or quantification, the specimen was re-reviewed with expert oversight until agreement was reached. After trained readers demonstrated sustained agreement with expert readers, they conducted slide examinations independently, with periodic expert supervision and quality checks. Each sample slide was first scanned at low power to systematically examine the smear, and areas or structures of interest were then examined at higher magnification. We used a 40x objective for confirmatory identification and count recording of ova and parasites, and this was repeated at least twice and often three times, with all recordings detailed on the laboratory data sheet. Three smear readings were performed whenever sufficient specimen was available, whereas two readings were completed when remaining sample volume was limited. All data were later entered into a master Excel database that included sample ID, fecal weight, and egg counts per smear for each type of parasite, noting any other characteristics observed. Work areas were disinfected with ethanol and bleach following each session, and waste was properly disposed of, in accordance with biosafety procedures.

### 2.4 Statistical analysis

Each parasite outcome was dichotomized into a binary variable specifying presence or absence, as the observed counts were highly right-skewed and zero-inflated (S2 Fig). We summarized parasite co-occurrence using pairwise associations among the binary variables, restricting analyses to pairwise complete cases. For each comparison, we computed the Jaccard similarity index (number of individuals with co-infection among individuals with either infection) to quantify the degree of co-occurrence while excluding joint absences, important for rare parasites [20]. We also computed the log odds ratio from pairwise 2x2 tables to measure the strength and direction of association, with a continuity correction of 0·5 to handle sparse cells [21], alongside Fisher’s exact test at α=0·05.

Parasite prevalences were estimated using Bayesian multilevel logistic regression models fitted separately for each outcome. All models included random intercepts to account for spatial variability in parasite prevalence and the nested structure of individuals within households, households within communities, and communities within regions. This accounts for region-, village-, and household-level clustering and borrows information across groups to increase estimation stability while allowing regions to contribute more equally.

To estimate overall and region-specific prevalences for each outcome, we fit intercept-only multilevel models including these random intercepts and no individual-level covariates. Overall estimates were computed as the inverse-logit of the population-level intercept with random effects set to zero, representing the predicted probability of infection for an individual in an average-risk region, village, and household under the fitted model. Region-specific prevalences were obtained from posterior expected probabilities for each region, computed as the inverse-logit of the overall intercept plus the region-level random intercept. These estimates reflect the observed demographic composition within each region and describe the total burden of infection as it occurs in the population.

To estimate age- and sex-specific prevalences, we fit multilevel models that additionally included fixed effects for age (categorized) and sex while retaining the same random intercept structure, with reference levels set to the categories with greater observations: female and 20–49 years of age. Age-sex specific prevalences were obtained from posterior expected probabilities for each age-sex combination after accounting for geographic clustering. Models including age-sex interaction terms were also included as sensitivity analyses but had poorer model fit.

All prevalence estimates were summarized using posterior means and 95% credible intervals. For soil-transmitted helminths, prevalence exceeding 20% was interpreted as indicative of endemicity according to World Health Organization criteria [22]. Each analysis used data from all individuals with available data on the outcome of interest, excluding those with missing measurements (S1 Table). For each model, four chains of 4,000 iterations were run, along with a burn-in period of 1,000 iterations and thinning to every third iteration. Convergence was assessed by inspection of effective sample sizes and potential scale reduction factors for all parameters [23]. Model fit was assessed with leave-one-out cross validation (S3 Table) and posterior predictive checks (S3 Fig). All analyses were performed in R version 4·3·1 [24] with RStan using the ‘brms’ package, using default non-informative and weakly informative priors [25]. Co-occurrence analyses additionally used the ‘vegan’ package [26].

## 3. Results

### 3.1 Sample characteristics

Of all 3,902 participants, the mean age was 18·6 (SD 16·5) years, with the southwest reporting the lowest mean age (16·4) and the northeast reporting the highest mean age (21·2). There were 1,767 (45·6%) males and 2,105 (54·4%) females, with the percentage male ranging from 41·5% in the southeast to 47·8% in the northeast. *H. nana* and *S. mansoni* were missing for 597 individuals in the northeast, while other helminths and *Entamoeba coli* were only available for these 597 individuals in the northeast, due to differences in the parasites examined in Golden et al. [16] compared to the other two studies (S1 Table).

All parasites exhibited heavy right skew in the distribution of intensity, particularly *A. lumbricoides*, *T. trichiura*, and *H. nana* (S2 Fig). Trends for the intensity of parasite infection generally paralleled trends for prevalence, with high-prevalence regions also reporting highest mean egg counts (Table 2).

**Table 2:** Sample population descriptive statistics by region. For each of the intestinal parasites, mean counts are provided, followed by the number of individuals with any non-zero quantity observed, as well as any missingness if applicable. Abbreviations: NE = northeast, SE = southeast, SW = southwest, WC = west coast, CP = central plateau. The dashed line means no summary statistic was calculated because there was no data available for that cell.

Variable	NE N = 1,357	SE N = 730	SW N = 662	WC N = 528	CP N = 595	OverallN = 3,872
Age in years: mean (SD)	21·2 (17·5)	18·5 (16·1)	16·4 (14·7)	17·3 (16·1)	16·6 (15·8)	18·6 (16·5)
Age group: n (%)						
< 2	98 (7·2%)	42 (5·8%)	42 (6·3%)	42 (8·0%)	46 (7·7%)	270 (7·0%)
2 - 4	160 (11·8%)	116 (15·9%)	112 (16·9%)	84 (15·9%)	102 (17·1%)	574 (14·8%)
5 - 11	311 (22·9%)	177 (24·2%)	179 (27·0%)	136 (25·8%)	160 (26·9%)	963 (24·9%)
12 - 19	198 (14·6%)	116 (15·9%)	104 (15·7%)	85 (16·1%)	104 (17·5%)	607 (15·7%)
20 - 49	469 (34·6%)	238 (32·6%)	200 (30·2%)	148 (28·0%)	152 (25·5%)	1,207 (31·2%)
≥ 50	121 (8·9%)	41 (5·6%)	25 (3·8%)	33 (6·3%)	31 (5·2%)	251 (6·5%)
Sex: n (%)						
Male	649 (47·8%)	303 (41·5%)	311 (47·0%)	243 (46·0%)	261 (43·9%)	1,767 (45·6%)
Female	708 (52·2%)	427 (58·5%)	351 (53·0%)	285 (54·0%)	334 (56·1%)	2,105 (54·4%)
*A. lumbricoides* (n = 3872)						
Mean (SD)	12·0 (51·7)	35·8 (92·0)	1·4 (11·1)	0·2 (2·6)	14·5 (60·1)	13·5 (57·0)
Any: n (%)	590 (43·5%)	427 (58·5%)	49 (7·4%)	6 (1·1%)	216 (36·3%)	1,288 (33·3%)
*T. trichiura* (n = 3872)						
Mean (SD)	1·14 (6·69)	11·89 (36·89)	1·05 (10·40)	0·00 (0·00)	0·12 (0·63)	2·84 (17·64)
Any: n (%)	375 (27·6%)	467 (64·0%)	44 (6·6%)	0 (0·0%)	44 (7·4%)	930 (24·0%)
Hookworm (n = 3872)						
Mean (SD)	0·21 (0·90)	0·04 (0·40)	0·01 (0·21)	0·00 (0·00)	0·00 (0·05)	0·08 (0·58)
Any: n (%)	186 (13·7%)	19 (2·6%)	7 (1·1%)	0 (0·0%)	1 (0·2%)	213 (5·5%)
*Strongyloides* (n = 3872)						
Mean (SD)	0·0129 (0·1750)	0·1053 (2·5203)	0·0038 (0·0582)	0·0025 (0·0458)	0·0059 (0·0737)	0·0263 (1·1000)
Any: n (%)	14 (1·0%)	13 (1·8%)	3 (0·5%)	2 (0·4%)	4 (0·7%)	36 (0·9%)
*H. nana* (n = 3275)						
Mean (SD)	0·0007 (0·0181)	0·0146 (0·1686)	2·1310 (16·2978)	0·6098 (5·5213)	0·2597 (2·3109)	0·5797 (7·7565)
Any: n (%)	1 (0·1%)	9 (1·2%)	80 (12·1%)	35 (6·6%)	20 (3·4%)	145 (4·4%)
*Missing*	597	0	0	0	0	597
*S. mansoni* (n = 3275)						
Mean (SD)	0·0020 (0·0314)	0·1543 (0·9968)	0·0020 (0·0303)	0·0028 (0·0486)	0·0249 (0·2228)	0·0403 (0·4846)
Any: n (%)	3 (0·4%)	49 (6·7%)	3 (0·5%)	2 (0·4%)	11 (1·8%)	68 (2·1%)
*Missing*	597	0	0	0	0	597
Helminths (n = 597)						
Mean (SD)	0·76 (0·77)	–	–	–	–	0·76 (0·77)
Any: n (%)	345 (57·8%)	–	–	–	–	345 (57·8%)
*Missing*	760	730	662	528	595	3,275
*E. coli* (n = 597)						
Mean (SD)	4·06 (30·48)	–	–	–	–	4·06 (30·48)
Any: n (%)	35 (5·9%)	–	–	–	–	35 (5·9%)
*Missing*	760	730	662	528	595	3,275

### 3.2 Co-occurrence

Co-occurrence patterns differed by parasite species. Jaccard similarity suggested strong co-occurrence between *A. lumbricoides* and other helminths, moderate co-occurrence between *A. lumbricoides* and *T. trichiura*, and weak co-occurrence between other helminths and both *T. trichiura* and hookworm (Fig 1). However, results with other helminths should be interpreted cautiously due to small sample size, as measurements were only available in a subset of participants from the northeast region. Patterns based on log odds ratios were broadly concordant with the Jaccard results, and additionally suggested strong positive associations between *Strongyloides* and *S. mansoni* and between *A. lumbricoides* and hookworm, as well as a moderate positive association between *S. mansoni* and *T. trichiura*. Because *Strongyloides* and *S. mansoni* prevalence was low, the elevated log odds ratio may reflect shared non-infection more than positive co-occurrence, as the Jaccard similarity was low. In contrast, *H. nana* showed moderate negative associations with both *A. lumbricoides* and *T. trichiura*. Notably, all individuals infected with other helminths were also co-infected with *A. lumbricoides*, *T. trichiura*, or hookworm (S2 Table).

### 3.3 Overall prevalence and geographical variation in intestinal parasite infection

Examining overall prevalences for the parasites (Table 3 and S4 Fig), we found that, among parasites measured for all regions, prevalence was highest for *A. lumbricoides* (22·0% [credible interval (CI) 2·8-67·3%]) and *T. trichiura* (15·3% [CI 0·4-68·9%]) and lowest for *Strongyloides (0·5% [CI 0·1-1·2%]) and S. mansoni* (0·5% [CI 0·0-1·9%]). Other helminths and *E. coli* were only measured in the northeast, but were observed at very high levels (54·2% [CI 3·2-97·7%] for other helminths and 24·3% [CI 0·3-93·6%] for *E. coli*). Unlike the crude proportions in Table 2, which may be affected by uneven sampling, these model-based prevalence estimates account for clustering in the data and provide a more stable summary of infection risk for an average-risk region, village, and household as estimated by the model.

**Table 3:** Estimated overall prevalence (%) of parasite presence across all sampled regions. Parasite presence is defined as a binary, set to 1 if any presence of the parasite was detected in the stool sample and 0 otherwise. Summaries are given in percentages for the mean, median, and 2·5% and 97·5% posterior interval bounds. *H. nana* and *S. mansoni* were not measured and therefore missing for 597 individuals in the northeast, while other helminths and *E. coli* were only available for these 597 individuals in the northeast.

Parasite	Mean	Median	2·5%	97·5%
*A. lumbricoides*	22·0	17·4	2·8	67·3
*T. trichiura*	15·3	8·4	0·4	68·9
Hookworm	2·5	0·6	0·0	20·2
*Strongyloides*	0·5	0·4	0·1	1·2
*H. nana*	4·7	2·4	0·3	24·3
*S. mansoni*	0·5	0·3	0·0	1·9
*Helminths*	54·2	56·7	3·2	97·7
*E. coli*	24·3	12·6	0·3	93·6

**Fig 2:**
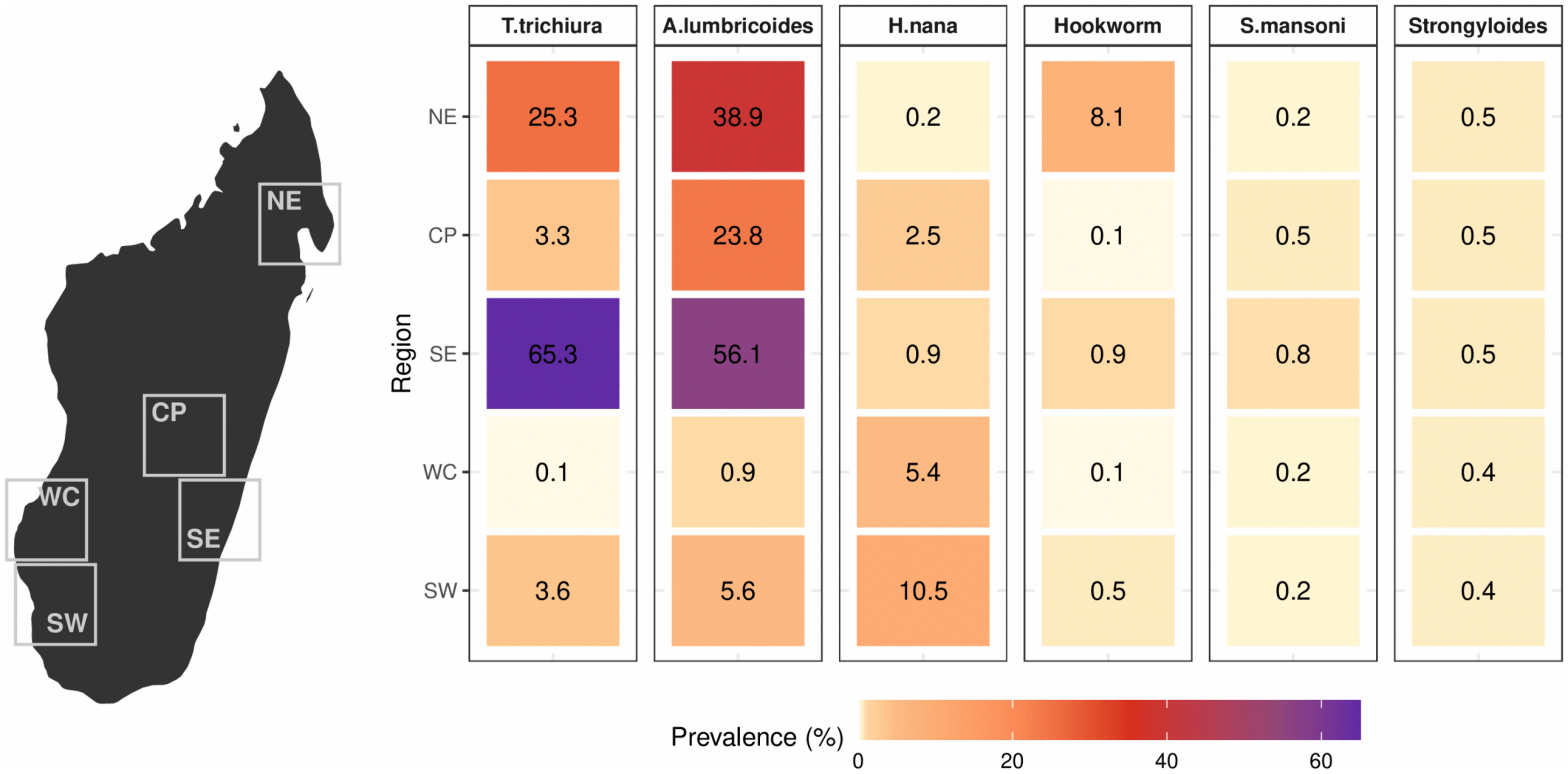
Observed variation in prevalence of intestinal parasites for all ages and sexes across regions of Madagascar. The relative difference in prevalence is shown as a heatmap with the color scale ranging from the minimum to maximum observed prevalence across all regions and parasite species. The prevalence (as a percentage) estimate is shown within each cell. Data are based on the first sample collected from each individual The prevalences of other helminths (54.2%) and *E. coli* (24.3%) were only assessed in the northeast region and are not presented in the heatmap. Base map source: Natural Earth public domain country boundary data (https://www.naturalearthdata.com/downloads/50m-cultural-vectors/50m-admin-0-countries-2/). Study-site points were plotted using the authors’ study data.

Geographical variation in prevalence was substantial for most parasites (Figs 2 and S5; S4 Table). There was a 600-fold difference between the lowest and highest regional prevalence of *T. trichiura*, an 80-fold difference in hookworm, a 60-fold difference in *A. lumbricoides*, and a 50-fold difference in *H. nana*. Only *Strongyloides* did not have any major regional variation in prevalence, with prevalences spanning 0·4% and 0·5% across all regions. Prevalence of *S. mansoni* was also relatively low across all regions, spanning from 0·2% (northeast, southwest, west coast) to 0·5% (central plateau) to 0·8% (southeast).

Parasite burden was generally highest in the northeast and southeast and lowest in the west coast. This was particularly true for soil-transmitted helminths (STH), with *A. lumbricoides* affecting over half of the population in the southeast (56·1%), over a third in the northeast (38·9%), and around a quarter in the central plateau (23·8%), occurring at endemic levels in three regions, with endemicity defined as at least 20% STH prevalence (World Health Organization, 2017). Levels of *T. trichiura* were similarly high, reaching 65·3% in the southeast and 25·3% in the northeast. Prevalence of hookworm was mostly found in the northeast (8·1%). The converse trend was observed for *H. nana*, which had the lowest prevalence in the northeast and southeast but had higher levels in the southwest (10·5%) and west coast (5·4%), both regions with much lower STH burden.

### 3.4 Variation in parasite infection by age and sex

**Fig 3:**
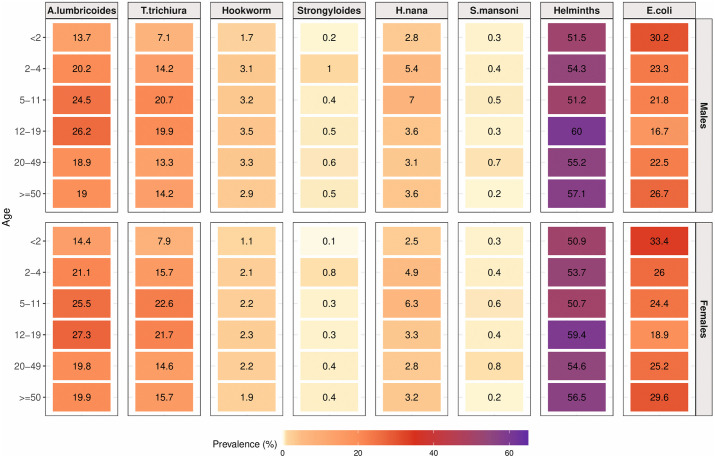
Estimated prevalence of intestinal parasites by age and sex. For each parasite, the relative difference in prevalence is shown as a heatmap with the color scale ranging from the minimum to maximum observed prevalence. The prevalence (as a percentage) estimate is shown within each cell. The prevalence statistics account for the nested spatial structure of sampling.

Examining trends by age, we see an approximately 2- to 3-fold difference in modeled prevalence across age groups by sex for soil-transmitted helminths (*A. lumbricoides*, *T. trichiura*, and hookworm), with prevalence generally highest for school-aged children aged 5–19 (Fig 3 and S6; S5 Table). The 12–19 age group had the highest prevalences for *A. lumbricoides* (26·2% for males; 27·3% for females), hookworm (3·5% for males; 2·3% for females), and other helminths (60·0% for males, 59·4% for females). The 5–11 age group had the highest prevalences for *T. trichiura* (20·7% for males, 22·6% for females) and *H. nana* (7·0% for males; 6·3% for females). *Strongyloides* was most prevalent in young children aged 2–4 years (1·0% for males; 0·8% for females), while *S. mansoni* was most frequently found in adults aged 20–49 (0·7% for males; 0·8% in females). Infants <2 years generally had lower prevalences compared to other age groups, with the exception of *E. coli*, where infection was highest for infants <2 years and the elderly 50 + population.

Differences between sex were minor for all parasites, with prevalence slightly higher in females than in males for *A. lumbricoides*, *T. trichiura*, *S. mansoni*, and *E. coli*, and the converse true for hookworm, *Strongyloides*, *H. nana*, and other helminths. The most noticeable difference was for hookworm, where prevalence in males was around 1·5-fold higher than in females for all age groups, with the highest prevalence occurring in the 12–19 age group for both sexes (3·5% in males and 2·3% for females). Models including an age-sex interaction yielded nearly identical prevalence estimates but showed slightly poorer leave-one-out cross-validation performance than the primary additive models (S3 Table; S7 and S8 Figs). Overall, it is clear that parasites vary far more by age than by sex.

## 4. Discussion

This study provides a comprehensive assessment of intestinal parasite prevalence across the lifecourse for both sexes in Madagascar, examining eight intestinal parasites across five climates and ecologically diverse regions. The highest parasite burden occurred in the humid rainforest regions of Mananjary (southeast) and Maroantsetra (northeast), where *A. lumbricoides* and *T. trichiura* prevalence far exceeded WHO endemicity thresholds [22]. Prevalence of other helminths and *E. coli* was also high in the northeast. This was consistent with high STH levels found in previous studies conducted in the eastern rainforest regions [10,11], although levels of hookworm were lower in our analyses. The eastern regions are some of Madagascar’s most rural and resource-limited areas. High levels of infection may be driven by the warm and humid climates that facilitate breeding of STH eggs, as well as increased contamination due to poor sanitation infrastructure [27]. Moisture is critical for the survival and development of helminth eggs and larvae in soil, with *A. lumbricoides* and *T. trichiura* eggs requiring moist soil to embryonate and hookworm larvae requiring moist conditions to be motile and contact human skin [27]. In contrast, dry conditions with high temperatures, low humidity, and intense sunlight can kill eggs and larvae, thereby reducing transmission.

Overall parasite burden was lower in Toliara (southwest), Morombe (west coast), and Fandriana (central plateau). In the hot and arid climate of the southwest and west coast, there is less fertile soil for STH transmission, which may explain the observed low prevalences (<6% in the southwest and <1% in the west coast) of *A. lumbricoides*, *T. trichiura*, and hookworm infections, though these values are lower than those reported in prior studies [9]. In contrast, *H. nana* prevalence was highest in these regions. Unlike STHs, *H. nana* transmission can occur person-to-person as well as through contaminated food and water. Few studies have examined *H. nana* prevalence in Madagascar; however, recent studies among school-aged children in the northern districts of Ambatoboeny, Mampikony, and Mahajanga reported respective prevalences of 9·1% [8], 0·4% [7], and 0·4% [6], all lower than the levels observed in the southwest in our analyses.

In addition to these climatic and soil-based features, WASH (water, sanitation, and hygiene) factors may also play a role in determining the relative prevalences of intestinal parasite infections across regions. In rural areas across the country, only approximately 36% of the population have access to improved water sources and only 10% of the population uses basic sanitation facilities [28], with roughly 45% of the population practicing open defecation [29]. The rural areas across the east coast (both Maroantsetra and Mananjary regions) are characterized by deficient WASH systems and are considered hazard prone, with flooding often compromising sanitation efforts. WASH systems are often considered the best in the central highland regions like the Fandriana region, whereas WASH systems in the western (Morombe) and southwestern (Toliara) regions are often water scarce and may face challenging sanitation issues. These social, infrastructural, and behavioral factors that can lead to differences in intestinal parasite prevalence were not the focus of the current research.

There was very little detected *Strongyloides* and *S. mansoni* infection (<1%) across all regions and age groups. Other studies conducted at sites elsewhere in the country have found much higher prevalences, with *Strongyloides stercoralis* prevalence in the east coast estimated to be 35·2% [10], and *S. mansoni* ranging from 5·0% across Western Madagascar [13] to 14·1-59·5% in the central plateau [9,30] and 73·6% in a remote Eastern district [12]. The low detection in our analyses may be due to low sensitivity of microscopy to light-intensity infections, particularly for these two parasites [31,32].

Though demographic differences were not as pronounced as geographic heterogeneity, there were some trends indicating potential at-risk groups. Consistent with previous literature, school-aged children and adolescents bore the highest burden of helminth infections, possibly due to greater soil and water contact during play and farming, greater likelihood of not wearing shoes, as well as incomplete development of immunity [11,33]. Sex differences were most noticeable for hookworm, where prevalence was higher in males for all age groups, which has been reported in previous studies [10].

The high observed prevalence of intestinal parasite infection, particularly of soil-transmitted helminths in the eastern regions, highlights the need for improved public health and policy action. The Malagasy government has been conducting twice yearly “health days” in all communities across the country where vitamin A supplements and deworming medication (400mg Albendazole to target soil-transmitted helminths) are provided to children under five years of age. Our results show that this intervention is not effective. Targeting interventions geographically will be critical given the ecological diversity of Madagascar and the high heterogeneity seen in parasite prevalences. The persistence of endemicity despite ongoing preventative chemotherapy and deworming programs [34] also suggests the importance of multi-sectoral and integrated approaches to address the broader socioeconomic and environmental drivers of infection. These include improvements to water, sanitation, and hygiene (WASH) education and infrastructure, adequate anthelminthic medicine access, and continued epidemiological monitoring of potential hotspots [11,33].

Interventions must also address the underlying drivers of poverty, food insecurity, and ecological instability in contributing to parasitic infections. Rising temperatures can impact the life cycles of many parasites, potentially increasing their prevalence in affected areas [35]. Other climate shocks such as cyclones, droughts, and heavy wind disrupt food systems, damage infrastructure, and facilitate disease transmission [36,37]. Given that the majority of the Malagasy population engages in subsistence agriculture, morbidity and disability due to parasitic infections can stifle economic growth and exacerbate already high levels of poverty. Micronutrient deficiencies, which can result from parasitic infections, have been found to be highly prevalent in the country [38] and are particularly concerning for women of reproductive age [33]. Policies to strengthen health systems, manage environmental risks for transmission, and improve the resilience of food systems are increasingly needed to address these challenges.

Though our study provides important geographically diverse results on multiple intestinal parasites, our findings should be interpreted in light of several limitations. First, parasite counts obtained by microscopy may underestimate true prevalence, particularly for light-intensity infections, and eggs may have been vulnerable to degradation during the transport process. Our study used ethanol for storage followed by freezing which is known to potentially degrade hookworm eggs, which may lead to some underestimation in our study. Further research using tests with higher sensitivity and specificity will be helpful for validating the results [39]. For example, although our methods are well suited for detecting various intestinal parasites, Kato-Katz techniques are more sensitive for detecting soil-transmitted helminths and S. mansoni and the Baermann method for S. stercoralis. Therefore, our research may underestimate the prevalence of both hookworm and Schistosoma. Second, the data do not comprise a representative sample of all regions in Madagascar. Though our survey included data from multiple regions, some areas remain underrepresented and not all parasites were measured in all regions. Future research may build upon our foundation to address these limitations through a more comprehensive data collection program that can provide nationally representative information on parasite prevalences. Third, data were collected at different timepoints and years and were analyzed cross-sectionally; therefore, analyses were not able to suggest causal relationships or capture temporal fluctuations related to seasonality. Extensions to our work may consider longitudinal analyses to better understand how social and ecological factors have influenced parasitic transmission in Madagascar over time.

## Supporting information

S1 Fig CONSORT diagram of study participants included in the analysis. Abbreviations: “MAHERY” refers to Golden et al. (2017); “Antongil” refers to Golden et al. (2019); “CRS” refers to Golden et al. (2020); SE = southeast, SW = southwest, WC = west coast.
(TIF)

S1 Table Number of individuals in each dataset with data available (subsetted to the first time point available for those with multiple measurements) for each intestinal parasite, including data for age and sex. (XLSX)

S2 Fig Distributions of intestinal parasites in the data, subsetting to the first available timepoint for each individual. (TIF)

S2 Table Parasite co-occurrence values in pairwise 2x2 tables, using all pairwise complete cases. Abbreviations: n00 = absence of both parasites; n01 = absence of parasite 1 and presence of parasite 2; n10 = presence of parasite 1 and absence of parasite 2; n11 = presence of both parasites; n = pairwise sample size.
(XLSX)

S3 Table Leave-one-out cross-validation (LOO-CV) model fit for all prevalence models, comparing the intercept-only models for overall prevalence (“Overall”), the models including age- and sex- fixed effects (“Age-Sex”), and the models with age-sex fixed effects and an age-sex interaction (“Age-Sex Int”). For each parasite, the three models are ranked by expected log predictive density (ELPD), where higher ELPD indicates better fit. The difference in ELPD (“ELPD Diff”) is reported relative to the best performing model. “SE Diff” refers to the standard error of the ELPD difference.
(XLSX)

S3 Fig Posterior predictive checks of the intercept-only Bayesian multilevel logistic regression models, for all intestinal parasite outcomes. (TIF)

S4 Fig Overall estimated prevalence for each parasite, computed by taking the inverse-logit of the global intercepts. Points represent the mean, crosses represent the median, and intervals represent the 95% uncertainty intervals.
(TIF)

S5 Fig Region-specific estimated probabilities of parasite presence. Points represent the median and intervals represent the 95% uncertainty intervals for each region.
(TIF)

S4 Table Region-specific estimated parasite prevalences. Mean estimates are displayed with 95% uncertainty intervals in parentheses. “Helminths” refers to other helminths not already displayed.
(XLSX)

S6 Fig Age-specific estimated parasite prevalences for males (top) and females (bottom). Points represent the median and intervals represent the 95% uncertainty intervals for each age category.
(TIF)

S5 Table Age-specific estimated parasite prevalences in percentages for males (M) and females (F). Mean estimates are displayed with 95% uncertainty intervals in parentheses. “Helminths” refers to other helminths not already displayed.
(XLSX)

S7 Fig Estimated prevalence of intestinal parasites by age and sex using models with age-sex interactions. For each parasite, the relative difference in prevalence is shown as a heatmap with the color scale ranging from the minimum to maximum observed prevalence. The prevalence (as a percentage) estimate is shown within each cell.
(TIF)

S8 Fig Age-specific estimated parasite prevalences for males (top) and females (bottom) using models with age-sex interactions. Points represent the median and intervals represent the 95% uncertainty intervals for each age category.
(TIF)

## References

[1] Chen J, Gong Y, Chen Q, Li S, Zhou Y. Global burden of soil-transmitted helminth infections, 1990–2021. Infect Dis Poverty. 2024;13(1):77. doi: 10.1186/s40249-024-01238-9 39444032 PMC11515461

[2] World Health Organization. Soil-transmitted helminth infections. World Health Organization. 2023. Accessed 2025 August 20. https://www.who.int/news-room/fact-sheets/detail/soil-transmitted-helminth-infections

[3] Akman M, Cebeci D, Okur V, Angin H, Abali O, Akman AC. The effects of iron deficiency on infants’ developmental test performance. Acta Paediatr. 2004;93(10):1391–6. doi: 10.1111/j.1651-2227.2004.tb02941.x 15499963

[4] Greigert V, Abou-Bacar A, Brunet J, Nourrisson C, Pfaff AW, Benarbia L, et al. Human intestinal parasites in Mahajanga, Madagascar: the kingdom of the protozoa. PLoS One. 2018;13(10):e0204576. doi: 10.1371/journal.pone.0204576 30304028 PMC6179227

[5] Habib A, Andrianonimiadana L, Rakotondrainipiana M, Andriantsalama P, Randriamparany R, Randremanana RV. High prevalence of intestinal parasite infestations among stunted and control children aged 2 to 5 years old in two neighborhoods of Antananarivo, Madagascar. PLoS Negl Trop Dis. 2021;15(4):e0009333. doi: 10.1371/journal.pntd.0009333 PMC8087024 33878113

[6] Razafiarimanga ZN, Yao YBK, Rajerison M, Randriamampianina LJ, Rahelinirina S, Rakotoarison R, et al. Risk factors for intestinal parasite portage in an informal suburb on the West coast of Madagascar. Parasite Epidemiol Control. 2022;19:e00267. doi: 10.1016/j.parepi.2022.e00267 36065443 PMC9440058

[7] Richert W, Kasprowicz D, Kołodziej D, Zarudzka D, Korzeniewski K. Intestinal parasitic infections among school children in northern Madagascar. Ann Agric Environ Med. 2024;31(4):546–51. doi: 10.26444/aaem/189514 39743713

[8] Richert W, Kołodziej D, Zarudzka D, Kasprowicz D, Świetlik D, Korzeniewski K. Intestinal parasites and hematological parameters in children living in Ambatoboeny District, Madagascar. Pathogens. 2024;13(11):930. doi: 10.3390/pathogens13110930 39599483 PMC11597123

[9] Tapia-Veloz G, Gozalbo M, Guirao V, Dinari H, Fuentes MV, Trelis M. Integrated evaluation of undernutrition, anaemia, and intestinal parasitic infections in school-aged children: a cross-sectional study in three regions of southern Madagascar. Children (Basel). 2025;12(8):990. doi: 10.3390/children12080990 40868442 PMC12384542

[10] Scarso S, Rakotoarivelo RA, Hey JC, Rasamoelina T, Razafindrakoto AR, Rasolojaona ZT, et al. Prevalence of Strongyloides stercoralis and other helminths in four districts of Madagascar. Trop Med Health. 2024;52(1):49. doi: 10.1186/s41182-024-00619-y 39075624 PMC11285119

[11] Hakami L, Castle PM, Kiernan J, Choi K, Rahantamalala A, Rakotomalala E, et al. Epidemiology of soil transmitted helminth and Strongyloides stercoralis infections in remote rural villages of Ranomafana National Park, Madagascar. Pathog Glob Health. 2019;113(2):94–100. doi: 10.1080/20477724.2019.1589927 30879406 PMC6502231

[12] Spencer SA, Penney JMSJ, Russell HJ, Howe AP, Linder C, Rakotomampianina ALD, et al. High burden of Schistosoma mansoni infection in school-aged children in Marolambo District, Madagascar. Parasit Vectors. 2017;10(1):307. doi: 10.1186/s13071-017-2249-7 28646926 PMC5483300

[13] Rasoamanamihaja CF, Rahetilahy AM, Ranjatoarivony B, Dhanani N, Andriamaro L, Andrianarisoa SH, et al. Baseline prevalence and intensity of schistosomiasis at sentinel sites in Madagascar: informing a national control strategy. Parasit Vectors. 2016;9:50. doi: 10.1186/s13071-016-1337-4 26822783 PMC4730633

[14] Krumkamp R, Remkes A, Hainasoa J, Rasamoelina T, Razafindrakoto AR, Razafindralava NM, et al. Prevalence of schistosome infection in a region of Madagascar regularly undergoing mass drug administration: a cross-sectional study. Pathog Glob Health. 2026;120(2):130–9. doi: 10.1080/20477724.2026.2616620 41623127 PMC13137748

[15] Kislaya I, Rakotoarivelo RA, Rasamoelina T, Solonirina J, Brito A, Ratiaharison EF, et al. Prevalence of schistosome infection among children under two years of age: a brief report from medium-to-high endemic regions of Schistosoma mansoni in Madagascar. Trop Med Health. 2025;53(1):179. doi: 10.1186/s41182-025-00871-w 41345890 PMC12679769

[16] Golden CD, Anjaranirina EJG, Fernald LC, Hartl DL, Kremen C, Milner Jr DA, et al. Cohort profile: The Madagascar health and environmental research (MAHERY) study in north-eastern Madagascar. Int J Epidemiol. 2017;46(6):1747–8. doi: 10.1093/ije/dyx071 29040632 PMC5837654

[17] Golden CD, Borgerson C, Rice BL, Allen LH, Anjaranirina EJG, Barrett CB, et al. Cohort description of the madagascar health and environmental research-antongil (MAHERY-Antongil) study in Madagascar. Front Nutr. 2019;6:109. doi: 10.3389/fnut.2019.00109 31428615 PMC6690017

[18] Golden CD, Rice BL, Randriamady HJ, Vonona AM, Randrianasolo JF, Tafangy AN, et al. Study protocol: a cross-sectional examination of socio-demographic and ecological determinants of nutrition and disease across Madagascar. Front Public Health. 2020;8:500. doi: 10.3389/fpubh.2020.00500 33042943 PMC7527467

[19] Centers for Disease Control and Prevention. Stool specimens – specimen processing. 2016. Accessed 2025 November 19. https://www.cdc.gov/dpdx/diagnosticprocedures/stool/specimenproc.html

[20] Jaccard P. The distribution of the flora in the alpine zone.1. New Phytol. 1912;11(2):37–50. doi: 10.1111/j.1469-8137.1912.tb05611.x

[21] Haldane JB. The estimation and significance of the logarithm of a ratio of frequencies. Ann Hum Genet. 1956;20(4):309–11. doi: 10.1111/j.1469-1809.1955.tb01285.x 13314400

[22] World Health Organization. Preventive chemotherapy to control soil-transmitted helminth infections in at-risk population groups. 2017. https://apps.who.int/iris/handle/10665/258983 29578660

[23] Gelman A, Rubin DB. Inference from iterative simulation using multiple sequences. Stat Sci. 1992;7(4):457–72. doi: 10.1214/ss/1177011136

[24] R Core Team. R: a language and environment for statistical computing. 2023. https://www.R-project.org

[25] Bürkner PC. brms: an R package for bayesian multilevel models using Stan. J Stat Softw. 2017;80(1). doi: 10.18637/jss.v080.i01

[26] Dixon P. Vegan, a package of R functions for community ecology. J Veg Sci. 2003;14(6):927–30. doi: 10.1111/j.1654-1103.2003.tb02228.x

[27] Sturrock SL, Yiannakoulias N, Sanchez AL. The geography and scale of soil-transmitted helminth infections. Curr Trop Med Rep. 2017;4(4):245–55. doi: 10.1007/s40475-017-0126-2

[28] Water, sanitation and hygiene. UNICEF. Accessed 2026 May 8. https://www.unicef.org/madagascar/en/programme/wash

[29] Department of Economic and Social Affairs. Madagascar. Accessed 2026 May 8. https://sdgs.un.org/basic-page/madagascar-34129

[30] Gruninger SK, Rasamoelina T, Rakotoarivelo RA, Razafindrakoto AR, Rasolojaona ZT, Rakotozafy RM, et al. Prevalence and risk distribution of schistosomiasis among adults in Madagascar: a cross-sectional study. Infect Dis Poverty. 2023;12(1):44. doi: 10.1186/s40249-023-01094-z 37098581 PMC10127445

[31] Schwarz NG, Rakotozandrindrainy R, Heriniaina JN, Randriamampionona N, Hahn A, Hogan B, et al. Schistosoma mansoni in schoolchildren in a Madagascan highland school assessed by PCR and sedimentation microscopy and Bayesian estimation of sensitivities and specificities. Acta Trop. 2014;134:89–94. doi: 10.1016/j.actatropica.2014.03.003 24657847

[32] Buonfrate D, Formenti F, Perandin F, Bisoffi Z. Novel approaches to the diagnosis of Strongyloides stercoralis infection. Clin Microbiol Infect. 2015;21(6):543–52. doi: 10.1016/j.cmi.2015.04.001 25887711

[33] World Health Organization. Schistosomiasis and soil-transmitted helminthiases: progress report, 2023. 2024. https://www.who.int/publications/i/item/WHO-WER9948-707-717

[34] WHO-AFRO. ESPEN - Madagascar. World Health Organization. 2025. Accessed 2025 September 8. https://espen.afro.who.int/maps-data/countries/madagascar#overview--introduction

[35] Short EE, Caminade C, Thomas BN. Climate change contribution to the emergence or re-emergence of parasitic diseases. Infect Dis (Auckl). 2017;10:1178633617732296. doi: 10.1177/1178633617732296 29317829 PMC5755797

[36] Rakotobe ZL, Harvey CA, Rao NS, Dave R, Rakotondravelo JC, Randrianarisoa J, et al. Strategies of smallholder farmers for coping with the impacts of cyclones: A case study from Madagascar. International Journal of Disaster Risk Reduction. 2016;17:114–22. doi: 10.1016/j.ijdrr.2016.04.013

[37] Harvey CA, Rakotobe ZL, Rao NS, Dave R, Razafimahatratra H, Rabarijohn RH, et al. Extreme vulnerability of smallholder farmers to agricultural risks and climate change in Madagascar. Philos Trans R Soc Lond B Biol Sci. 2014;369(1639):20130089. doi: 10.1098/rstb.2013.0089 24535397 PMC3928894

[38] Golden CD, Zamborain-Mason J, Levis A, Rice BL, Allen LH, Hampel D, et al. Prevalence of micronutrient deficiencies across diverse environments in rural Madagascar. Front Nutr. 2024;11:1389080. doi: 10.3389/fnut.2024.1389080 38826583 PMC11140575

[39] Mejia R, Vicuña Y, Broncano N, Sandoval C, Vaca M, Chico M, et al. A novel, multi-parallel, real-time polymerase chain reaction approach for eight gastrointestinal parasites provides improved diagnostic capabilities to resource-limited at-risk populations. Am Soc Trop Med Hyg. 2013;88(6):1041–7. doi: 10.4269/ajtmh.12-0726 PMC3752800 23509117

